# Microbiological Evaluation of 5 L- and 20 L-Transparent Polypropylene Buckets for Solar Water Disinfection (SODIS)

**DOI:** 10.3390/molecules24112193

**Published:** 2019-06-11

**Authors:** M. Inmaculada Polo-López, Azahara Martínez-García, Maria Jesus Abeledo-Lameiro, Hipolito H. Gómez-Couso, Elvira E. Ares-Mazás, Aurora Reboredo-Fernández, Tracy D. Morse, Lyndon Buck, Kingsley Lungu, Kevin G. McGuigan, Pilar Fernández-Ibáñez

**Affiliations:** 1 CIEMAT-Plataforma Solar de Almería, 04200 Tabernas, Almería, Spain; amartinez@psa.es; 2 Laboratory of Parasitology, Department of Microbiology and Parasitology, Faculty of Pharmacy, University of Santiago de Compostela, 15782 Santiago de Compostela, Spain; mariajesus.abeledo@usc.es (M.J.A.-L.); hipolito.gomez@usc.es (H.H.G.-C.); melvira.ares@usc.es (E.E.A.-M.); aurora.reboredo.fernandez@usc.es (A.R.-F.); 3 Institute of Food Research and Analysis, University of Santiago de Compostela, 15782 Santiago de Compostela, Spain; 4 Department of Civil and Environmental Engineering, University of Strathclyde, Glasgow G1 1XQ, UK; tracy.thomson@strath.ac.uk; 5 School of Art, Design and Performance, Buckinghamshire New University, High Wycombe HP11 2JZ, UK; lyndon.buck@bucks.ac.uk; 6 Department of Environmental Health, University of Malawi, Malawi; klungu@poly.ac.mw; 7 Department of Physiology and Medical Physics, Royal College of Surgeons in Ireland (RCSI), DO2 YN77 Dublin, Ireland; kmcguigan@rcsi.ie; 8 NIBEC, School of Engineering, Ulster University, Newtownabbey BT37 0QB, Northern Ireland, UK

**Keywords:** drinking water, household water treatment and storage, SODIS, *E. coli*, MS2-phage, *Cryptosporidium*

## Abstract

Background: Solar water disinfection (SODIS) is an appropriate technology for household treatment of drinking water in low-to-middle-income communities, as it is effective, low cost and easy to use. Nevertheless, uptake is low due partially to the burden of using small volume polyethylene terephthalate bottles (1.5–2 L). A major challenge is to develop a low-cost transparent container for disinfecting larger volumes of water. (2) Methods: This study examines the capability of transparent polypropylene (PP) buckets of 5 L- and 20 L- volume as SODIS containers using three waterborne pathogen indicators: *Escherichia coli*, MS2-phage and *Cryptosporidium parvum*. (3) Results: Similar inactivation kinetics were observed under natural sunlight for the inactivation of all three organisms in well water using 5 L- and 20 L-buckets compared to 1.5 L-polyethylene-terephthalate (PET) bottles. The PP materials were exposed to natural and accelerated solar ageing (ISO-16474). UV transmission of the 20 L-buckets remained stable and with physical integrity even after the longest ageing periods (9 months or 900 h of natural or artificial solar UV exposure, respectively). The 5 L-buckets were physically degraded and lost significant UV-transmission, due to the thinner wall compared to the 20 L-bucket. (4) Conclusion: This work demonstrates that the 20 L SODIS bucket technology produces excellent bacterial, viral and protozoan inactivation and is obtained using a simple transparent polypropylene bucket fabricated locally at very low cost ($2.90 USD per unit). The increased bucket volume of 20 L allows for a ten-fold increase in treatment batch volume and can thus more easily provide for the drinking water requirements of most households. The use of buckets in households across low to middle income countries is an already accepted practice.

## 1. Introduction

Despite many efforts to increase access to drinking water, more than two billion people rely on either unimproved water sources or faecally contaminated improved sources [1]. Every year, half a million people die from preventable diarrheal diseases particularly in low-to-middle-income countries. The 2030 UN Sustainable Development Agenda calls for universal access to safe drinking water. Therefore, development of sustainable and affordable point-of-use water treatment technologies to deliver safe drinking water at the household level is a priority [2].

The primary criteria for household water treatment and safe storage (HWTS) technologies is that they should be effective (in providing safe water), available, affordable and acceptable to the most vulnerable (children under five, immune-compromised, affected by emergencies and outbreaks, etc.) [2]. Efficacy can be measured through the inactivation or removal of reference water pathogens (bacteria, virus and protozoa). The bacterium *Escherichia coli* is sensitive to disinfection, frequently found in untreated surface waters and is used as indicator of faecal contamination. It is the third most frequently reported pathogen responsible for childhood diarrhoea [3]. Coliphages are proposed as surrogates for human enteric viruses, which are the smallest pathogens. They show widely varying susceptibilities to water treatments and have high infectivity, for example rotavirus is the most frequently reported water pathogen in relation to childhood diarrhoea [4]. The highly infectious oocysts of *Cryptosporidium parvum*, are least sensitive to inactivation by chemical disinfection [5] and are recognised as the second most frequently reported pathogen responsible for childhood diarrhoea [6].

Existing household water treatment and storage (HWTS) technologies include boiling, filtration, chemical disinfection, coagulation/flocculation, UV-C disinfection and solar disinfection. All have been recently evaluated following the International Scheme to Evaluate HWTS Technologies [7]. Membrane ultrafiltration is the most effective in terms of reduction of all pathogens, followed by chlorination, but these have the significant limitation of high cost (membrane ultrafiltration), adverse taste and potential generation of disinfection by-products when the water is turbid (chlorination).

Solar water disinfection (SODIS) consists of filling transparent containers with biologically contaminated water and exposing them to direct sunlight for at least 6 h [8]. SODIS is considered to be an appropriate HWTS technology for treating drinking water in low-to-middle-income communities for the following reasons:

1. Effectiveness: against a wide range of waterborne pathogens [8].
2. Cost: low-cost or zero-cost of the technology in areas where transparent containers (typically PET bottles) are available.
3. Ease of Use: it can be employed by any user with very little training.
4. Appropriateness: SODIS uses available sunlight to reduce the microbial load of water without using any chemical additives, high technology or electrical supply.

SODIS is a practical, sustainable and affordable intervention used in many parts of the world where access to safe drinking water is problematic and available solar radiation levels are high (global solar irradiance > 1800 kWh/m^2^ per year).

SODIS inactivates microbial organisms via a combination of: (1) solar UV-B; (2) solar UV-A radiation, oxidative activity associated with dissolved oxygen and other endogenous components in the cells and (3) thermal conditions during solar exposure [9]. PET bottles are the most frequently used SODIS containers since they are readily available at minimal cost in most countries. PET bottle SODIS has been tested in Africa, Latin America and Southeast Asia [8]. Its disinfection performance has been assessed for a number of water pathogens, i.e., *E. coli, Salmonella, Vibrio cholerae, Enterococcus faecalis*, MS2-phage, norovirus, hepatitis A virus, and *C. parvum* [8,10]. SODIS has been rigorously assessed under laboratory conditions, i.e., controlled solar irradiance, temperature and chemical and biological composition of water, and in the field, under variable conditions of irradiation, temperature, mixed cultures of wild pathogens, and chemical parameters, i.e., dissolved and suspended organic and inorganic matter [8].

Despite the proven efficacy of SODIS under real conditions, significant obstacles to uptake remain. Some studies have shown engineered approaches to improve SODIS efficacy, including increase of solar UV radiation input entering the photo-reactor by using compound parabolic collectors [11]. Enhancement by adding small amounts of hydrogen peroxide or iron at micro- to milli-molar levels, has also been reported [12].

Recent research has focused on increasing the daily volume of water treated by SODIS. Several recent solar reactor designs (dynamic flow-through or static) have been shown to treat volumes in the range between 2 to 50 L per day using various transparent materials, geometries, mirrors, etc. The capability of these systems for water disinfection has been assessed under natural sunlight against several pathogens, *E. coli*, *E. faecalis*, MS2-phage, bacterial spores, and *C. parvum* oocysts [13,14]. Sophisticated solar reactor designs are more efficient for pathogen removal with higher outputs of water but less appropriate for HWTS in low-income communities owing to their high cost of manufacture.

Recent low-cost, simple, and non-technological SODIS developments have achieved impressive disinfection results. SODIS bags made of low-density polyethylene (LDPE) is one such example [15]. However, the bags gradually degrade under sunlight after 12 weeks, and some organic chemicals (2,4-Di-tert-butylphenol, at levels of 1–4 μg/L) were detected leaching from the plastic at concentrations above the disinfection by-products limits established by the Environmental Protection Agency [16]. Others evaluated bags were made of food-grade polyethylene (PE) joined with layers of bio-oriented-polypropylene (PP), polyamide and PET. Improved designs of 4 L SODIS bags obtained very good disinfection results for *E. coli* (6-Log Reduction Value (LRV) in 60 min) and 5 months durability in the PE bags [17]. The use of large volume transparent containers made of polycarbonate has also been explored. Despite low UV transmission (<50%) 19 L water cooler bottles demonstrated good SODIS efficacy for water containing *E. coli* in both Europe and Asia [18]. Therefore, exploring large volume ‘traditional’ containers could be a fruitful approach to expanding the use of SODIS as they are already in use in the community, they are also affordable and they are accepted by the end-user.

This work focused on Malawi within a large research project (EU H2020 WATERSPOUTT Project) developing solar enhanced water treatment technologies in four African countries [19]. Although, Malawi reports 87% safe drinking water access coverage [20], as elsewhere in Africa, a major problem is the disparity in access to safe drinking water between urban and rural environments, where 19% and 81% of the population reside, respectively. Household water treatment has been reported in only 31% of Malawi households, with the majority in rural areas. The methods used were chlorination (64%), boiling (28%), filtration (9%) and natural settlement (17%) [20]. In addition, there has been a significant shift in the type of containers used for water collection and storage in rural households. The Malawi arm of the WATERSPOUTT study was based in the Chikwawa District, in southern Malawi, where there is a steady increase in the use of plastic buckets (7.7% in 2013 to 58% in 2017) due to increased availability and affordability [19,21]. These lidded buckets also help reduce post collection contamination of water within the household. Many rural households in Malawi have one or more buckets in use within the household for a variety of purposes, including water collection.

This study reports the evaluation of transparent polypropylene (PP) buckets as SODIS containers. Malawi-manufactured 5 L- and 20 L-buckets were tested under full natural sunlight for periods of 6 h. Three waterborne reference pathogens were used, *E. coli*, MS2-phag, and *C. parvum*, according to the protocol for testing HWTS [7]. Ageing of the containers was also investigated.

## 2. Materials and Methods

### 2.1. Transparent PP Buckets

**Figure 1:**
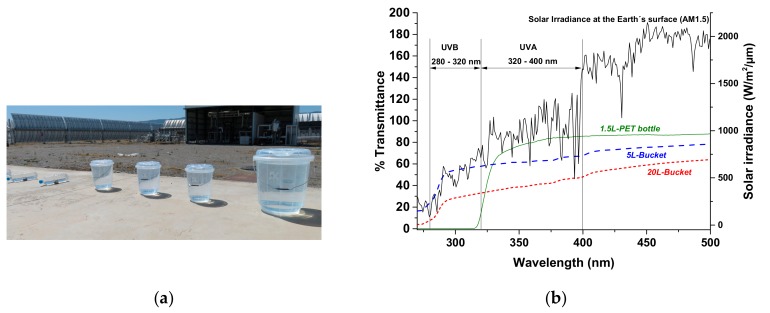
5 L- and 20 L polypropylene transparent buckets and 1.5 L -PET bottles (**a**) placed at Plataforma Solar de Almeria (PSA) facilities under natural solar radiation. (**b**) Comparison of transmittance (λ: 250 to 600 nm) of the three SODIS containers and the local solar UV irradiance measured during one of the solar tests.

Transparent PP lidded buckets (5 L- and 20 L-buckets with lids) were used for this work and were compared with 1.5 L-PET bottles (Figure 1a). Pieces of 2 × 2 cm from each container were cut and the optical transmittance (from 260 to 600 nm) was measured using a UV-spectrophotometer (Thermo Unicam II, Spectronic CamSpec Ltd., Leeds, UK); results are shown in Figure 1b. Buckets were produced by ArKay Plastics, Ltd. (Blantyre, Malawi) from nucleated random PP copolymer for injection moulding applications. Metrics for the 5 L- and 20 L-buckets and lids are, respectively: total weight (160 g and 886 g), wall thickness (0.95 and 1.60 mm), height (22.6 cm and 32.8 cm), diameter (19.4 cm and 30.8 cm), illuminated surface calculated as the cross surface area in the horizontal plane (0.043 m^2^ and 0.101 m^2^) and 1% by weight UV stabilizer (1.6 g and 8.86 g). The researchers asked the manufacturer (Arkay plastics, Ltd.) to increase the UV-stabilizer from the previous level of 0.5% by weight of plastic to 1% by weight to provide a greater level of UV resistance without adversely affecting the UV transmittance for SODIS.

### 2.2. Well Water Description

All experiments used water from a 200 m depth borehole well located at Plataforma Solar de Almeria (PSA) in Spain. Naturally occurring organisms in well water were below the detection limit (2 colony-forming units per mL, CFU/mL) determined by Endo agar standard plate count techniques. Turbidity was measured using a turbidity meter (Hach-2100N, Loveland, CO, USA). Total Organic Carbon was determined using a TOC analyser (Shimadzu TOC-5050, Japan). Iron concentration in the water samples was determined by UV-spectrophotometry using the ISO 6332. The main properties of the well water were, pH 7.8; turbidity = 1.5 NTU; TOC = 5 mg/L; and iron = 0.05 mg/L.

### 2.3. Water Pathogens

#### 2.3.1. *E. coli* Strain and Enumeration

*E. coli* K12 (American Type Collection Culture ATCC 23631, Manassas, VA, USA) was used for all tests. Enumeration and quantification method used are described elsewhere [11]. The strain was inoculated from stocks in 14 mL of Luria broth nutrient medium (Miller’s LB Broth, Sigma-Aldrich, Gillingham, UK) and incubated at 37 °C at constant agitation under aerobic conditions. After 18 h the bacteria were in the stationary phase at a concentration of 10^9^ CFU/mL. Bacterial suspensions were centrifuged at 800× *g* for 10 min and the pellet was then re-suspended in 14 mL PBS (Phosphate Buffer Saline, 0.01 M phosphate buffer, 0.0027 M potassium chloride and 0.137 M sodium chloride, pH 7.4; Sigma-Aldrich). Appropriate dilution was made directly into the water containers to obtain initial concentration of 10^6^ CFU/mL. The standard plate count method was used to enumerate the bacterial cells during tests. 10-fold serial dilution of the most concentrated samples in PBS and volumes of 20 µL in triplicate were added on Endo agar (Sigma–Aldrich) for *E. coli* enumeration. When the bacterial concentration was low enough to be enumerated in drops of 20 µL, 500 µL aliquots of samples were spread on the same agar dishes to reach a detection limit (DL) of 2 CFU/mL. Colonies were counted after 24 h of incubation at 37 °C.

#### 2.3.2. MS2 Strain and Analysis

MS2 Coliphage (ATCC 15597B1) and the bacterial host *E. coli* C300 (ATCC 15597) were obtained from the ATCC (Manassas, VA, USA). Stock culture preparation and propagation of MS2 infective particles were conducted using tryptone glucose yeast (TYG) extract agar containing the following reagents from Sigma-Aldrich: Tryptone (10.0 g/L) Yeast Extract (1.0 g/L), NaCl (8.0 g/L), Glucose (10.0 g/L), CaCl_2_ (2.94 g/L) and Thiamine (0.1 g/L). Enumeration of infective MS2 was determined by a double-layer agar method and expressed as plaque-forming units per mL (PFU/mL). Briefly, 1 mL of sample, 0.1 mL of *E. coli* host suspension and 5 mL of melted semi-solid TYG agar are mixed and poured on petri dishes containing TYG agar. After the TYG broth layer had solidified, petri dishes were incubated at 35 °C for 18 h. DL of this method was 1 PFU/mL.

#### 2.3.3. *Cryptosporidium parvum* Oocysts and Viability Assays

*Cryptosporidium* oocysts were collected from naturally infected neonatal Friesian-Holstein calves. Protocols for concentration in PBS (Sigma-Aldrich), purification (discontinuous caesium chloride gradients (Sigma-Aldrich)), quantification (Neubauer haemocytometer) and molecular characterisation are reported elsewhere [22]. Briefly, faeces were collected from calves by rectal sampling and stored at 5 °C. Faecal material was homogenised with 10–20 mL of PBS (0.04 M, pH 7.2), filtered through two sieves (mesh sizes 150 and 45 µm), shaken with diethyl ether (2:1, *v*/*v*) and concentrated by centrifugation at 2000× *g*, 4 °C, for 15 min. The resulting uppermost two layers were carefully discarded and the sediment was washed with PBS by centrifugation at 2000× *g*, 4 °C, for 15 min. *Cryptosporidium* oocysts were purified on discontinuous caesium chloride gradients of 1.05, 1.10 and 1.40 g/mL by centrifugation at 2000× *g*, 4 °C, for 30 min. Finally, the oocysts were counted in a modified Neubauer haemocytometer using 0.16% malachite green solution (Sigma-Aldrich) as counterstain. The isolate was characterized as *C. parvum* by the analysis of a ≈ 587 bp fragment of the SSU-rDNA (small subunit ribosomal RNA) gene [23].

The viability of *C. parvum* oocysts was determined by fluorogenic vital dye propidium iodide (PI) inclusion/exclusion with a further modification that includes an immunofluorescence antibody test to verify oocyst identification [22]. Briefly, every 2 h, different volumes of samples (1-5 L) were taken and filtered through nitrocellulose membranes (pore size, 2 µm) employing the concentration tube of the Filta-Max^®^ equipment (IDEXX Laboratories Inc., Westbrook, USA). The membranes were removed, placed in re-sealable polyethylene bags (Minigrip^®^, IDEXX Laboratories Inc.) and washed three times with 5 mL of PBS. The samples were centrifuged at 2000× *g* for 15 min and 200 µL of the sediments were incubated with 15 µL of PI (Sigma-Aldrich) working solution (1 mg/mL in PBS (0.1 M, pH 7.2)) and 15 µL of monoclonal antibodies labelled with fluorescein isothiocyanate (FITC) (Aqua-Glo^TM^ G/C Direct Test, Waterborne^TM^ Inc., USA) at 37 °C, for 30 min. Samples were then washed three times in PBS at 10,000 g, 4 °C, for 5 min. Oocysts were identified first under FITC filter (excitation at 450–480 nm; barrier at 515 nm) before being examined for PI inclusion/exclusion under a PI filter (excitation at 510–550 nm; barrier at 590 nm). The proportions of ruptured (ghost), PI-positive (dead), and PI-negative (viable) oocysts were quantified in an epifluorescence microscope equipped with a Nomarski differential interference contrast, FITC and PI filters (Eclipse 50i, Nikon, Tokyo, Japan). The results are shown as the percentage of PI-negative (viable) oocysts (Equation (1)), which were determined for each experiment after triplicate counts of more than 100 oocysts.

Oocyst viability (%) = (PI-Negative oocysts/Total oocysts) × 100(1)

### 2.4. Solar Experiments

All experiments were carried out for 5–6 h under completely sunny conditions in PSA (Almeria, Spain; latitude 37.0947° N, longitude 2.3584° W, altitude 500 m). At the start of the experiments (10:30–11:00 a.m. local time) the UV irradiance was around 20–25 W/m^2^ and increased during the experiments up to a maximum ~50 W/m^2^. Containers were filled with well water and suspensions of each microorganism were spiked into the water in separate experiments to achieve 10^6^ CFU/mL for *E. coli*, 10^5^ PFU/mL for MS2, and 2–5 × 10^6^ purified oocysts/L of *C. parvum*. After agitation for homogenisation in the dark, initial samples (t = 0 min) were taken and the containers were exposed to sunlight. Samples were taken regularly throughout each experiment to measure the variation of the cell/viral particles or oocyst viability and analysed as described. Dark samples were kept in the lab at room temperature (25 °C) and were analysed at the end of each experiment for control purposes. As expected, the dark control results showed stable microorganism concentrations (data not shown). All assays were performed in triplicate simultaneously for each microbial indicator (*E. coli*, MS2, *C. parvum*) and container (PP or PET) under natural sunlight.

Temperature (Checktemp, Hanna, Spain), was measured during the experiments. UV radiation was measured with a global UV pyranometer (295–385 nm, CUV4, Kipp & Zonen, Delft, The Netherlands) with a typical sensitivity of 264 µV W^−1^ m^−2^ which records diffuse and direct components of the global solar radiation in units of W/m^2^. Equation (2) was used to calculate the total UV energy dose received per unit of illuminated surface, where t is the experimental time and UV(t) is the measured solar UV irradiance.

UV-Dose = ∫UV(t)dt(2)

### 2.5. Ageing Tests

Degradation of the PP bucket material was analyzed under accelerated and natural conditions. Four 2 × 2 cm pieces were cut from each type of PP bucket lid. Plastic degradation was measured by transmittance scanning from 270 to 600 nm with a UV-spectrophotometer with an integrating sphere of 150 mm diameter and specular reflectance at incidence angles from 0° to 68° (Perkin-Elmer Lambda 1050, Beaconsfield, UK). Results correspond to the average values measured from all four pieces. Accelerated ageing was performed according to ISO-16474 in a chamber (Atlas UV Test, Atlas Materials Testing Technologies, Mt. Prospect, IL, USA). Briefly, it consists of the uninterrupted exposure of the pieces of material to 45 W/m^2^ of direct solar UV radiation, at 60 °C for 4 h and condensation at 50 °C for another 4 h. Transmittance of PP samples was measured (after cleaning with soft tissue) at regular intervals: 0, 300, and 600 h. This test was selected to expose the materials under extreme climate conditions according to the ISO standard for materials ageing. 

Natural ageing tests were performed by exposing pieces of plastic to the elements for 6 uninterrupted months (from January to September 2017) at PSA, in the Tabernas desert (Almeria, Spain). Spectral transmittance of the samples was measured after 0, 3, and 6 months of exposure time. The pattern of radiation during the tests conformed to typical profiles for winter-spring-summer months in the South of Spain. The average UV irradiance was measured continuously during these tests using the solar pyranometer described above. UV irradiance values at noon (max. in the day) ranged from 21.0 W/m^2^ in January, 36.6 W/m^2^ in March, 48.4 W/m^2^ in May, and 39.6 W/m^2^ in August.

### 2.6. Statistical Analysis

Differences in SODIS and dark control microbe viability were compared by pairwise multiple comparison procedures (Student-Newman-Keuls method) and one-way ANOVA using GraphPad Prism v5 Statistical Software (GraphPad Software, Inc., La Jolla, CA, USA). Differences were considered significant at *p* < 0.05. No significant differences were found in the triplicate results. The results presented in the graphs are the average of three replicates with the error bar representing standard deviation.

## 3. Results

### 3.1. Solar Disinfection Efficiency Within PP Buckets

#### 3.1.1. *E. coli*

**Figure 2:**
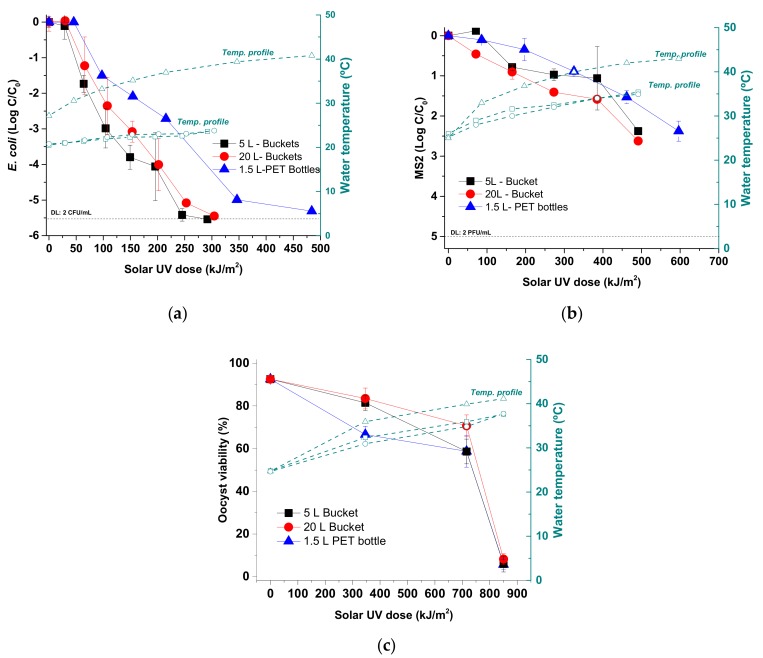
Solar inactivation of (**a**) *E. coli*, (**b**) MS2 coliphage and (**c**) *C. parvum* oocysts in 5 L- and 20 L-buckets against 1.5 L-PET bottles under natural sunlight. Water temperature profile is shown on the right Y-axis for all experiments.

Figure 2a shows the inactivation profile of *E. coli* K-12 under natural sunlight with PP buckets against 1.5 L-PET bottles. In all cases, the detection limit (DL = 2 CFU/mL) was reached, indicating ≥5.5 *E. coli* LRV in <4 h of solar exposure. The maximum irradiance recorded was 28 and 38.5 W/m^2^ for PP buckets and PET bottles, respectively.

No significant differences were observed for solar UV dose required to achieve the same inactivation result for 5 L- and 20 L. In both cases, around 250–300 kJ/m^2^ or 3 h of solar exposure were required to reach DL. In contrast, 500 kJ/m^2^ or 4 h of solar exposure was required to achieve the same results in the case of 1.5 L-PET bottles.

#### 3.1.2. MS2-Phage

Figure 2b shows the inactivation profile of MS2 coliphage under natural sunlight within 5 L- and 20 L-buckets against 1.5 L-PET bottles. Maximum solar UV irradiance recorded was 31 and 38.2 W/m^2^ for PP buckets and 1.5 L -PET bottles, respectively. 

No significant differences in terms of solar inactivation of this microorganism between the buckets or containers were observed. Reductions of 2.4 and 2.6 LRV were obtained in 5 L- and 20 L -buckets, respectively, requiring 500 kJ/m^2^ of solar UV dose (5 h). However, a higher solar UV dose (600 kJ/m^2^) was needed for PET bottles to achieve similar results (2.4 LRV).

#### 3.1.3. *C. parvum*

Initial oocyst viability of *C. parvum* isolate used in the experiments was 93.21 ± 1.34%. The maximum UV irradiance recorded within this period was 51.6 W/m^2^. Maximum temperatures reached in 1.5 L-PET bottles and 5 L- and 20 L-buckets were 42.5 °C, 39.4 °C and 38.6 °C, respectively (Figure 2c). No significant differences between temperatures recorded in 5 L- and 20 L-buckets were observed. However, temperatures registered in PET bottles were higher (by up to 5.6 °C) than those found in the buckets. This temperature variation has been proven not to have a significant effect in SODIS efficacy, this will be only of relevance at T > 45 °C [24].

After 2 h of natural solar exposure, oocyst viabilities observed in PET bottles were lower than the corresponding values observed in 5 L- and 20 L-buckets, showing statistically significant differences between them (66.60 ± 3.80% vs. 81.44 ± 3.57% and 83.50 ± 4.88%, respectively, *p* < 0.0001). The oocyst viability decreased further after 4 h and statistically significant differences were not detected between PET bottles and in 5 L-buckets (58.75 ± 7.42% and 58.64 ± 5.81%, respectively), but there were differences between PET bottles and 20 L-buckets (70.68 ± 5.09%, *p* < 0.0001). However, after 6 h of exposure (850 kJ/m^2^ solar UV dose), statistically significant differences were not observed in the percentage of viable oocysts among the three containers evaluated (5.68 ± 2.46%; 5.92 ± 3.73%; 8.23 ± 2.53% for PET bottles, 5 L- and 20 L-buckets, respectively) (Figure 2c).

### 3.2. Plastics Ageing and Transmission Properties of Buckets

**Figure 3:**
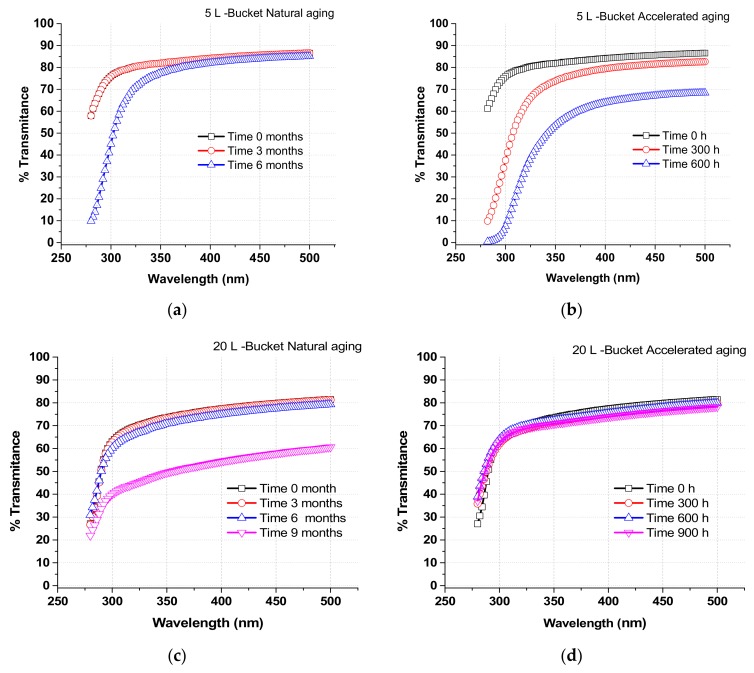
Transmittance of bucket walls. (**a**) 5 L bucket natural aging; (**b**) 5 L bucket accelerated aging; (**c**) 20 L bucket natural aging; (**d**) 20 L bucket accelerated aging. Time 0 months’ symbol is sometimes overlapped with ‘Time 3 months’ symbol.

Figure 3 shows comparative results of 5 L- and 20 L-buckets exposed to natural and accelerated solar ageing. UV transmission of the 5 L-bucket material reduced strongly after 6 months (natural) or 600 h (accelerated). On the other hand, the 20 L-bucket retained stable transmission properties under the same ageing conditions. The high level of degradation in the 5 L-bucket became obvious when the material was physically degraded (data not shown) after 9 months under natural environment. This result can be most likely attributed to the different wall thickness of 5 L- and 20 L-buckets of 0.95 and 1.60 mm, respectively.

## 4. Discussion

The results of this study demonstrate similar inactivation kinetics under natural conditions of solar radiation for *E. coli*, MS2-phage and *C. parvum*, in well water using 5 L- and 20 L-buckets compared to those observed in 1.5 L-PET bottles, which are the usual container used for SODIS. Therefore, the studied transparent PP buckets represent a good alternative to PET, allowing for the treatment of larger volumes of water.

McGuigan et al. [24] suggested that reactor volume may affect the SODIS efficacy as optical penetration would vary with diameter of the reactor and changes in temperature of the water will depend on container volume. Nonetheless, these authors did not observe any significant differences in the dynamics of inactivation of *E. coli* K-12 exposing different volumes of water (0.5 and 1.5 L) to solar radiation. However, on comparing the results obtained in a study using a 2.5 L-reactor fitted with Compound Parabolic collectors (CPC) and those obtained with a 25 L-reactor fitted with CPC with the same concentration factor of the solar radiation, Gómez-Couso et al. [13] found that the volume of water exposed to solar radiation had a negative effect on the efficacy of the technique. Recently, Keogh et al. [18] demonstrated that 19 L transparent polycarbonate water containers were as effective for inactivating *E. coli* as 2 L-PET bottles under natural sunlight in three different geographical areas (Spain, Bahrain and India). Similarly, the present work demonstrates the efficacy to inactivate several pathogen indicator organisms in 5 L- and 20 L-PP buckets.

It is well accepted that the SODIS disinfection mechanism consists of a series of exogenous and endogenous photo-activated multi-step chemical processes that result in inactivation of microorganisms. The UV component of the solar spectrum is responsible for the photo-inactivation processes, with shorter wavelengths (in the UV-B) being the most germicidal. Solar UV-B accounts for only a small fraction (ca. 5%) of total solar UV. Nevertheless, other mechanisms attributed to the UV-A region have been recognized to induce germicidal effects. In summary, these are a series of oxidative reactions produced by internal reactive oxygen species (ROS) photo-generated by UV-A photons that are absorbed by existing chromophores. ROS can oxidize most of the components and produce lethal damage from the formation of pyrimidine dimers, the peroxidation of proteins and lipids, reduction of membrane permeability and/or DNA rupture generating single strand breaks (SSBs) [25,26].

Previous studies report that UV-B radiation is approximately 100–1000 times more lethal for microorganisms than UV-A [27]. It is well known that the UV-B germicidal effect is mainly based on the direct effect on DNA stability as it overlaps with the DNA absorption region. UV-B absorption therefore favours the generation of genomic alterations, so-called DNA photo-products whose accumulation inhibits normal cell activity and ultimately leads to cell death. In addition, other internal cell components like proteins, chromophores compounds, enzymes, vitamins, and acids have been widely reported to be altered by the UV-B absorption [28].

The key to explaining the different efficiency of the evaluated plastic containers lies in their UV optical transmission properties. According to the transmittance of the materials used (PP in two different thickness values and PET) and the spectral solar irradiance, we can calculate the ratio of the UV-A and UV-B energy flux inside the containers (Table 1). PET bottles are essentially opaque to UV-B radiation and only transmit 61% of the total solar UV received, which is all UV-A. In contrast, both 5 L-PP and 20 L-PP containers transmit 11% and 6% of the total solar UV-B radiation, respectively. This explains why PP has better disinfection results compared to PET. The difference in transmittance between PP containers is attributed to their thicknesses, 0.95 mm (5 L) and 1.60 mm (20 L). When we calculate the UV photon-flux (W) as a function of the surface area of the container and normalized against the global UV inside each container (Table 1, right side), we observe that both PP containers have a similar fraction of UV-B (14.5% and 17.5%), which accounts for the very similar inactivation profile observed in Figure 2 for *E. coli* and MS2, and are more efficient than PET 1.5 L bottle whose UV-B is filtered out. The different volumes (5 L and 20 L) of both buckets could have an effect on light extinction due to the water path length in both containers (19.4 and 30.8 cm, respectively). Following the recent results by Castro et al. [14], when the water turbidity is zero, this factor is negligible, therefore in this case, volume had no significant effect on the disinfection efficiency as shown in our results [14].

As SODIS is used under natural conditions of strong sunlight, deterioration of the container is a potential problem. For this reason, PET bottles should be replaced every 6 months [29]. The 20 L-PP bucket could be used for up to 6 months under natural sunlight because the transmission properties remain stable. In contrast, transmission properties of the 5 L-bucket were strongly affected under the same conditions, suggesting that this container would need to be changed every 3 months.

The biocidal effect of SODIS is caused by UV inactivation, thermal heating, and synergy between both processes at temperatures above 45 °C [24,30]. Therefore, water temperature increase is a key factor for SODIS. During our study the water temperatures inside the PP buckets were significantly lower than corresponding values in PET bottles. The maximum temperatures observed ranged between 25 °C and 40 °C reached in the 5 L- and 20 L-PP buckets, respectively, and 43 °C inside the 1.5 L-PET bottles. Considering previous studies and the thermal profile observed in the buckets, with temperatures lower than 40 °C, this factor can be discarded as the main cause of pathogen inactivation [14,30]. However, in the case of *Cryptosporidium*, in previous SODIS studies carried out under field conditions, spontaneous excystation was observed [10]. Excystation of a small percentage of the sporozoites of *C. parvum* may occur when the oocysts are incubated at 37 °C in the absence of any other stimulus, which makes their survival within the bucket impossible because the environment is different from that provided by the typical host [31]. Moreover, solar radiation can cause sporozoite membrane depolarization, which results in reduced cellular ATP and increased cytosolic calcium, causing apical organelle discharge and a false start to regulated exocytosis [32]. In the present study, high percentages of spontaneously excysted oocysts were observed both in 1.5 L-PET bottles and in 5 L- and 20 L-PP buckets, reaching minimum values of 17.89%, 5.83% and 7.50% (2 h) and maximum values of 86.96%, 80.62% and 83.93% (6 h), respectively (data not shown).

In contrast, the fluorogenic vital dye technique used to evaluate the potential oocyst viability indicates the integrity and permeability of cellular membranes, which may be affected during SODIS. This technique often overestimates the potential infectivity of oocyst population compared with the neonatal murine model. Therefore, actual infectivity may be lower than reported oocyst viability [33]. Similar reductions in infectivity were also reported by Smith et al. [34] for partially SODIS inactivated *Shigella typhimurium*.

Transparent buckets have a high chance of being accepted as a point-of-use HWTS treatment in low-to-middle income countries across Sub-Saharan Africa. Existing high levels of plastic bucket ownership, demonstrates willingness to invest and use this technology for collection and storage. Use of transparent PP containers for treatment adds value to the existing product, and reduces time and money needed for alternative commonly used point-of-use water treatment options (e.g. boiling, chlorination). The buckets tested were locally produced for the same price as the standard opaque 20 L-buckets available in country. The 20 L-PP bucket system could also address a current barrier to use of SODIS, whereby households would not have to fill a large number of 2 L-PET SODIS bottles therefore reducing the time and effort needed to provide safe drinking water to the whole household. The longevity of the transparent bucket in tests indicates a lifespan equivalent to that of current plastic buckets within the household (up to six months) dependent on the consistency of exposure. Further research is ongoing to evaluate acceptance of the technology at household level, taking into consideration consistency of use, trust and acceptability of households, accessibility and management of the technology (e.g. theft of buckets, access of animals and children during treatment). The chemical stability and potential toxicological implications of these materials under sunlight is key for future deployment of these systems in the communities. This on-going research goes beyond the main objective of the present paper.

Any HWTS technology developed must be aware of the local economic environment and take into consideration psychosocial issues that may affect use. As such, the speed of water treatment, volume that can be treated, unit cost and the container being used for treatment should take into consideration day to day use and current acceptance. For Malawi, average household size is 4.3 members who require a minimum of 80 L of water per day for drinking, bathing and cooking [1]. With this in mind, it is imperative that large batch volumes of water can be treated. The treatment method must be cost effective over sustained periods. With an average monthly income of €18 per month in the study area, any water treatment choice would have to ensure minimal cost within the household. The PP buckets described here were produced locally in Malawi at a commercial cost of $2.90 for a 20 L size. Controlled testing infers that these systems should last for a minimum of 6 months which equates to $0.01 USD per 20 L of water. This equates to the same cost as PET 2 L bottles (*n* = 10), which would be significantly more labour intensive and require a larger surface area to place bottles for concurrent treatment. Comparatively, chlorination of the same unprotected water using commercially available treatment would cost the same $0.01 per 20 L, but would result in a change of taste to the water, which has been reported as less preferred, require more regular investment rather than the one off cost of a bucket, and require improved compliance as previous studies have shown less than 50% of households use sufficient chlorine to make water safe for consumption [35].

The novelty and innovative aspect of this study is that the results demonstrate that the 20 L transparent PP SODIS bucket technology produces excellent bacterial, viral and protozoan inactivation and is obtained using buckets that were fabricated locally at very low cost ($2.90 USD per unit). The increased bucket volume of 20 L allows for a ten-fold increase in treatment batch volume (compared to the standard 2 L-PET SODIS bottle) and can thus more easily provide for the drinking water requirements for most households. The use of buckets in household across low to middle income countries is an already accepted practice.

## 5. Conclusions

Polypropylene 20 L buckets are more robust and more UV photo-stable than 5 L-PP buckets. UV induced deterioration of the thinner 5 L-buckets demonstrates that they would not be suitable for SODIS use under field conditions, whereas the 20 L containers would be suitable. Transparent 20 L-PP buckets are highly effective for solar disinfection of bacterially contaminated (*E. coli*) water. Reductions of >5 LRV (99.999%) to below the limit of detection are achieved after 3 h of solar exposure. Reductions of 2.4 and 2.6 LRV of MS2-phage, under 500 kJ/m^2^-solar UV (5 h); and from 93.21 ± 1.34% to 5.92 ± 3.73% and 8.23 ± 2.53% *C. parvum* oocysts viability, under 850 kJ/m^2^-solar UV (6h) were observed in the 5 L- and 20 L-buckets, respectively. Similar inactivation kinetics to inactivate three waterborne pathogen indicator organisms in well water using 5 L- and 20 L-buckets with respect to 1.5 L-PET bottles were observed under natural conditions of solar radiation. Therefore, the PP buckets represent a good alternative to the PET bottles usually employed in SODIS procedures, allowing for the treatment of larger water volumes. This work provides reliable evidence that low-cost PP-transparent lidded buckets may be used for low-cost drinking water disinfection, via SODIS, as they are as efficient as recommended PET bottles for the reduction of microbial indicators, are higher volume (20 L), low-cost and already accepted by the community (i.e., Malawi).

## Figures and Tables

**Table 1:** Constituent solar energy components after transmission through the container wall expressed in percentage.

	Ratio–Transmitted Energy Flux (W/m^2^): Solar Input (W/m^2^) (%)			Area (m^2^)	Ratio–Spectral Fraction in a Range (W): Total Flux in the Container (W) (%)		
	UV (280–400 nm)	UV-B (280–320 nm)	UV-A (320–400 nm)		UV (280–400 nm)	UV-B (280–320 nm)	UV-A (320–400 nm)
**Solar**	**100**	20	80	-			
**PET-1.5L**	61	0	61	0.024	**100**	0.4	99.6
**PP-5L**	61	11	50	0.043	**100**	17.5	82.5
**PP-20L**	38	6	32	0.101	**100**	14.6	85.4

Note: The energy flux (W/m^2^) was calculated using the reference solar spectral irradiance: air mass 1.5 (ASTM G-173, American Society for Testing and Materials) for the different ranges of the UV spectrum and the transmission data of the different materials (Figure 1).
